# The Role of Evidence in the Decision-Making Process of Selecting Essential Medicines in Developing Countries: The Case of Tanzania

**DOI:** 10.1371/journal.pone.0084824

**Published:** 2014-01-08

**Authors:** Amani Thomas Mori, Eliangiringa Amos Kaale, Frida Ngalesoni, Ole Frithjof Norheim, Bjarne Robberstad

**Affiliations:** 1 Centre for International Health, Department of Global Public Health and Primary Care, University of Bergen, Bergen, Norway; 2 School of Pharmacy, Muhimbili University of Health and Allied Sciences, Dar es Salaam, Tanzania; 3 Ministry of Health and Social Welfare, Dar es Salaam, Tanzania; National Taiwan University, Taiwan

## Abstract

**Background:**

Insufficient access to essential medicines is a major health challenge in developing countries. Despite the importance of Standard Treatment Guidelines and National Essential Medicine Lists in facilitating access to medicines, little is known about how they are updated. This study aims to describe the process of updating the Standard Treatment Guidelines and National Essential Medicine List in Tanzania and further examines the criteria and the underlying evidence used in decision-making.

**Methods:**

This is a qualitative study in which data were collected by in-depth interviews and document reviews. Interviews were conducted with 18 key informants who were involved in updating the Standard Treatment Guidelines and National Essential Medicine List. We used a thematic content approach to analyse the data.

**Findings:**

The Standard Treatment Guidelines and National Essential Medicine List was updated by committees of experts who were recruited mostly from referral hospitals and the Ministry of Health and Social Welfare. Efficacy, safety, availability and affordability were the most frequently utilised criteria in decision-making, although these were largely based on experience rather than evidence. In addition, recommendations from international guidelines and medicine promotions also influenced decision-making. Cost-effectiveness, despite being an important criterion for formulary decisions, was not utilised.

**Conclusions:**

Recent decisions about the selection of essential medicines in Tanzania were made by committees of experts who largely used experience and discretionary judgement, leaving evidence with only a limited role in decision-making process. There may be several reasons for the current limited use of evidence in decision-making, but one hypothesis that remains to be explored is whether training experts in evidence-based decision-making would lead to a better and more explicit use of evidence.

## Introduction

Insufficient access to essential medicines is a major health challenge in developing countries; among poor populations more than half have been estimated to lack regular access to medicines [1]. Shortages of essential medicines are common in publicly-financed facilities, which constitute a major part of the health systems in most developing countries [2], [3], and which are especially important for poor families seeking affordable services [4]. Commonly mentioned problems are insufficient public spending on pharmaceuticals, the high cost of medicines and challenges in the supply chains [3], [4]. Efforts to improve access to essential medicines have been revitalised by the Millennium Development Goals [5] and a renewed global focus on Primary Health Care [6].

The essential medicines programme entails stocking a limited range of efficacious, safe and cost-effective medicines that are sufficient to meet the priority health needs of the people [7]. For many countries, essential medicines are those recommended in their treatment guidelines [8]. Consistent and appropriate use of adequately developed treatment guidelines and formularies improve the availability and use of medicines [8], [9], [10], and their effective implementation not only increases efficiency in resource use but also improves access and the overall quality of care [11], [12], [13].

### The Essential Medicines Programme in Tanzania: Historical Perspective

Tanzania, one of the pioneers of the essential medicines programme, produced its first list of essential medicines in the early 1970s [14], [15]. The programme was later adopted by the WHO, and in 1977 the first WHO modal list of essential drugs was produced [16]. In 1978, the provision of essential medicines was declared to be one of the key elements of Primary Health Care through the Alma Ata Declaration [17]. In 1990, Tanzania produced a national health policy document for the first time, adopting the Primary Health Care approach as its cornerstone strategy [18]. A year later, the country launched its first Standard Treatment Guidelines and National Essential Medicine List (STG/NEML) [19], which has subsequently been revised three times. The STG contains recommendations about appropriate healthcare decisions for common disease conditions in Tanzania and the NEML specifies the type of medicines and level of healthcare facility for which they should be made available. The NEML is also used to guide the procurement and supply of medicines in the public sector [11].

### Tanzanian Healthcare System

Tanzania is categorised as a low-income country with a per capita expenditure on health of about 41 US$ per year [20]. The healthcare system has a pyramid structure, with tertiary facilities at the apex and primary facilities at the base; in between these lie the regional and district facilities. The Government owns about three-quarters of all healthcare facilities, while the rest are private, with some belonging to faith-based organizations [21]. As in other sub-Saharan African countries, the burden of disease is dominated by infectious diseases [22] and about 60 per cent of medicines listed as essential have been estimated to be available in district and primary facilities [23]. Indicators show that the Tanzanian health system is facing large challenges, including a relatively low life expectancy and relatively high infant, child and maternal mortality rates (Table 1).

**Table 1:** Selected demographic and health indicators for Tanzania.

Indicator	Data	Source
Population	44.9 million	Census, 2012 [24]
Life expectancy at birth	52	TDHS*, 2010
Fertility rates	5.4	TDHS, 2010
Infant mortality rate	51/1,000	TDHS, 2010
Under five mortality rate	81/1,000	TDHS, 2010
Maternal mortality rate	454/100,000	TDHS, 2010 [25]

TDHS: Tanzania Demographic Health Survey.

### Essential Medicine Selection in Developing Countries

Most developed countries have health technology assessment (HTA) systems, such as the National Institute of Clinical Excellence (NICE) in the UK and the Canadian Agency for Drugs and Technology in Health (CADTH), which issue formulary recommendations for reimbursement decisions. Cost-effectiveness evaluation is a mandatory criterion employed to inform decision-making [26], [27]. By contrast, essential medicines in developing countries, including Tanzania, are selected by expert committees that supposedly use the WHO’s guidelines, which recommend selection to be based on evidence of efficacy, safety, cost and cost-effectiveness [28]. However, the extent to which the evidence-based approach has been implemented in developing countries is not well documented. Therefore this study aims to describe the process of updating the Standard Treatment Guidelines and National Essential Medicine List in Tanzania and further examines the criteria and underlying evidence used in decision-making.

## Methods

### Ethics Statement

The study was approved by the Ethical Review Committee of the Tanzania National Institute of Medical Research. The study was conducted during a period when there was a medical doctors’ strike in the country and therefore we anticipated challenges in obtaining written consents. This concern was communicated to the ethics committee and authorisation was granted to use verbal consent. After self-introduction, the purpose of the study was explained to each informant and confidentiality was assured. All the informants were nevertheless, cautiously, asked for written consent before commencing the interviews, but they opted to give verbal consent. Each informant was assigned a code number which was entered on a consent form and signed to document that verbal consent had been given. Furthermore, the interviews were recorded with permission from the informants and the digital voice recorder and the transcripts were kept confidential.

### The Study Design

This qualitative study utilises a descriptive case study design, which is an empirical inquiry that investigates a phenomenon within its real-life context [29]. This design is useful when studying complex and context-dependent undertakings, such as the selection of essential medicines [30]. The descriptive design was chosen in order to provide information-rich explanations of the decision-making processes [29], [31]. The study adheres to the consolidated criteria for reporting qualitative research (COREQ) [32].

### The Research Team

The research team consisted of two doctoral students, a senior researcher and two professors. ATM’s background is in pharmacy, health policy analysis and management. FN’s background is in medicine (MD) and health economics. EAK is a senior researcher (PhD) and has outstanding experience with the ATLAS.ti® data analysis software. OFN and BR are professors with extensive experience of national guidelines and drug reimbursement advisory committees in Norway.

### Sampling and Sample Size

We used purposive sampling methods to select 18 information-rich informants from the list of experts who participated in the revision of the STG/NEML and two who did not participate, but who were perceived to possess important information for the study. We obtained this list, which contained the names, professions, specialisations, institutions and phone and email contacts, from the Ministry of Health and Social Welfare (MoHSW). Some informants were contacted through phone calls while others were visited at their work places. All the selected informants agreed to participate in the study. Several other informants were also involved in the study through informal interviews which were conducted in order to broaden our understanding of the inquiry.

### Descriptions of Study Participants

**Figure 1:**
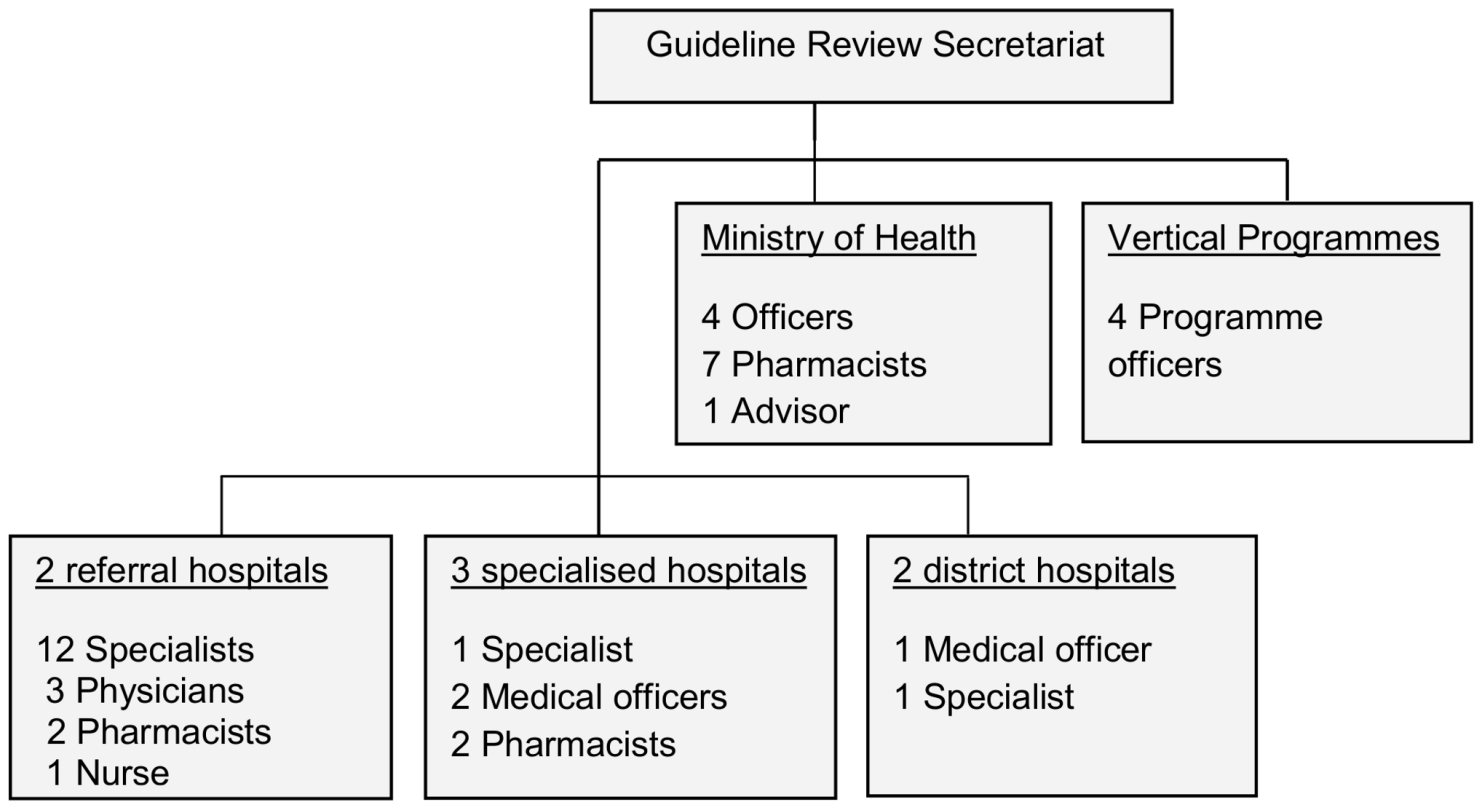
Summary of the key milestones during the revision process.

In selecting the informants, we chose those who had experience of participating in the previous revision process, but we also wanted to have good professional, institutional and speciality representations. Therefore our informants were pharmacists and clinicians with different specialisations from referral, municipal and specialised hospitals. Others were programme and section officers from the MoHSW (Figure 1). The final two informants were from the Food and Drugs Regulation Authority (TFDA). Eight of the 20 key informants were females working in hospitals, the MoHSW and the TFDA. Some of the participants, particularly the pharmacists, knew the interviewer in person.

### Data Collection Methods

In-depth interviews and document reviews were the main methods of data collection and were carried out between June and December 2012, while the revision of the STG/NEML was still ongoing.

#### In-depth interviews

In-depth interviews were conducted face to face with key informants, in English, using a pre-tested, semi-structured interview guide. All formal interviews were conducted in the offices of our informants and nobody else was present during the conversations. Interviews were digitally recorded and each lasted for 30–45 minutes. The 18 formal interviews, including one repeat interview, with the STG/NEML review group and the two additional in-depth interviews with informants from the TFDA were sufficient for data saturation. In addition, some informal interviews were conducted without the interview guide.

#### Document reviews

Several documents containing information related to the implementation of the essential medicines programme in Tanzania were reviewed to supplement the interview data. This included the STG/NEML of 2007 and 2012, the national drug policy, minutes and proceedings of the review meetings, published reports and research articles. We reviewed the malaria and HIV/AIDS treatment guidelines in order to determine whether they are consistent with international guidelines. The research team was already in possession of some of these documents, which were used for a systematic review study about the use of pharmacoeconomics as a criterion of medicine selection in Tanzania [33].

### Interview Guide

The interview guide contained questions and probes as described below. The flow varied from one participant to another depending on how the discussions unfolded. It was piloted with four participants and, because no major changes were introduced in the guide, we decided to include these interviews in the analysis.

#### The process of medicine selection

Informants were asked to describe how the review process was conducted and probed about how they became involved, how and by whom they were contacted and their personal views about the process. Those from the MoHSW who initiated and co-ordinated the process were in addition probed about how they selected the participants, the rationale for doing the review, composition of the committees etc.

#### Criteria for medicine selection

Informants were asked about how they selected the medicines, the selection criteria, how strictly the criteria were followed and to rank the criteria based on their importance/strengths. For each criterion they mentioned, they were probed to give the type and source of evidence and how they evaluated such evidence.

#### Use of economic evaluation evidence

Informants were asked whether they used economic analysis as a criterion if they had not mentioned it before, and how and to what extent economic evaluation was used (probed to give examples). They were further probed about challenges that hinder the use of economic evaluation and enabling factors for its use. Lastly they were asked if they had received any training in health economics.

### Data Management and Analysis

Verbatim data were transcribed into text using a standardised transcription protocol [34]. Transcripts were loaded into ATLAS.ti 7 Qualitative Data Analysis Software and analysed using a thematic content approach [35]. Each transcript was read carefully to identify relevant segments of text, which were then coded. Similar or related codes were organised into categories. Quotations attached to the codes were read with constant comparisons and the main descriptions were summarised in memos. Data from the interviews and document reviews were triangulated in memos. Finally, categories were organised under their respective pre-defined themes.

### Description of the Coding Tree

The coding tree consisted of three branches (themes); process, criteria and evidence. Under process there were two categories; the STG/NEML review and the approval process. Under criteria we did not have categories but only the codes for each criterion. Evidence was categorised as being drawn from experience and scientific study or from official documents. Code names reflected the content of each text segment.

### Data Validity

Five measures were taken to ensure that the data obtained were valid and trustworthy. Firstly, informants were contacted informally to create a platform for self-introduction, to explain the purpose of the research and assure confidentiality. Secondly, informants were purposively selected to maximise representation of a wide range of perspectives on the subject. Thirdly, interviews were recorded and then transcribed shortly after each interview session to preserve the originality of the data. Fourthly, transcripts were shared with the informants for cross-checking and validation, after which ten of the 18 informants provided feedback, most of them without any changes and a few with minor editing. Fifthly, triangulation of data was performed to enrich and supplement the information collected by interviews and document reviews.

## Results

This section provides results about the STG/NEML revision process, the criteria applied and the extent to which evidence was utilised during decision-making. To illustrate the findings, supporting verbatim quotes from the informants are provided. Informants are only identified by their institutions in order to avoid any breach of confidentiality.

### Description of the Revision Process

**Figure 2:**
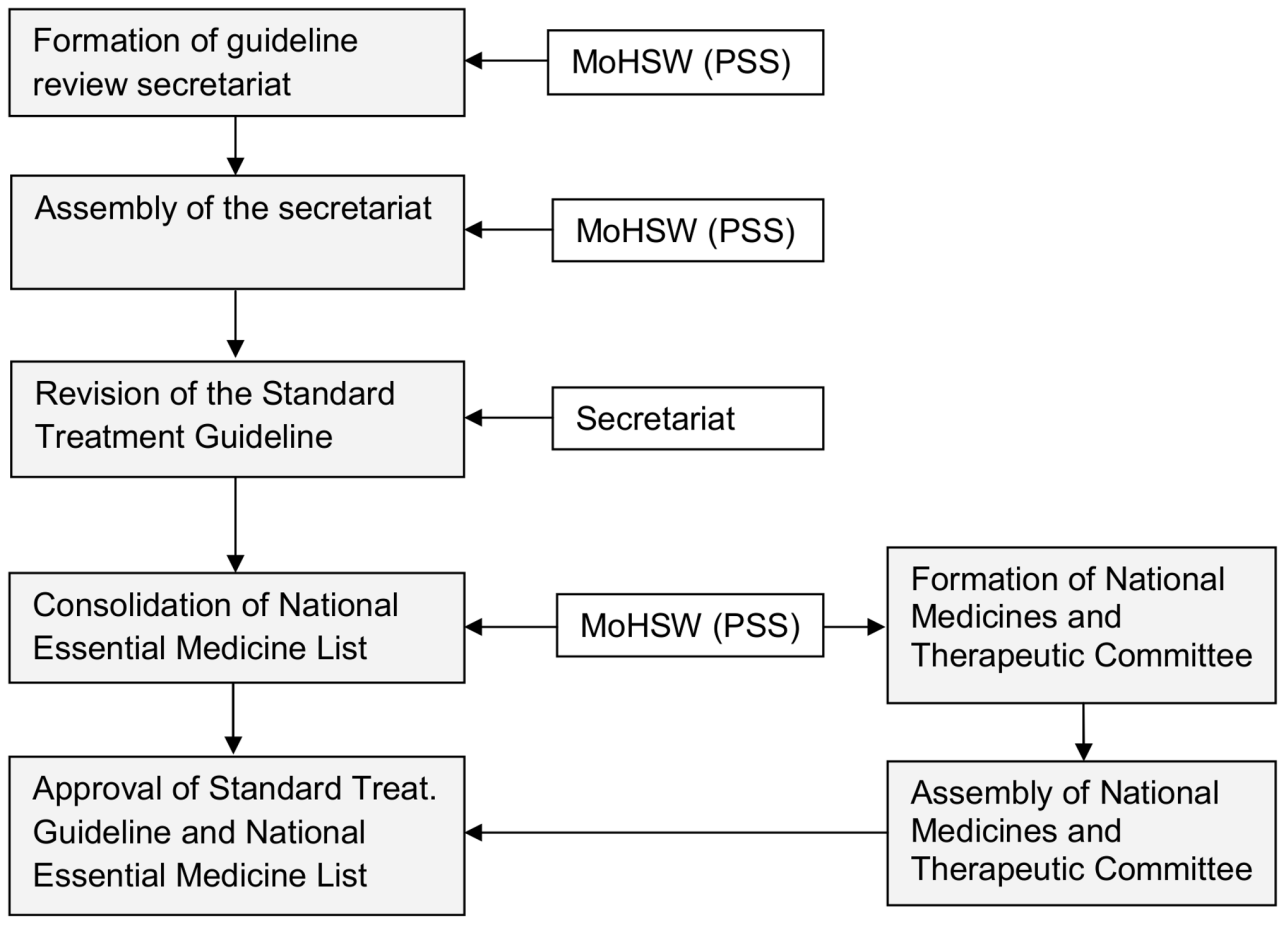
Composition and institutional representation of the guideline review Secretariat.

The process of updating the STG/NEML was initiated in early 2012 by the Ministry of Health and Social Welfare (MoHSW). The process was co-ordinated by the Pharmaceutical Service Section (PSS) on behalf of the National Medicines and Therapeutic Committee (NMTC). The sequence of events is shown in Figure 2. An official from the MoHSW gave the following rationale for the revision.

*The review was caused by two important things: first the number of diseases had increased and the required medicines to manage such diseases were not in the STG/NEML. Also it has been a long time since the existing STG/NEML was revised and there have been some new developments and changes in how certain diseases are managed in clinical practice. Basically, the WHO recommends revision after every 2–3 years*. (Participant from the MoHSW)

To begin the review process, the PSS convened an internal meeting to establish a committee of experts, known as ‘a guideline review secretariat’ to revise the STG first; this was important because only those medicines recommended in the STG are listed in the NEML. One official explained:

*First we had an internal meeting where we decided the kind of people to involve in the process; we wanted a mixture of people from primary to tertiary-level facilities, including people from various programmes. Therefore we consulted people from the malaria and HIV/AIDS control programmes who had the experience of reviewing their treatment guidelines and they gave us guidance about who we should involve in the review process*. (Participant from the MoHSW)

The STG/NEML document has two parts; the STG part contains 25 chapters covering common diseases in Tanzania, their clinical signs and symptoms, how they should be diagnosed and the recommended treatments or supportive care. The NEML part contains the list of all medicines that are recommended in the STG. It uses generic names and the medicines are arranged according to their pharmacological groups. The NEML also specifies the dosage form, its strength and the level of healthcare facilities where each medicine should be made available.

#### The guideline review secretariat

The guideline review secretariat was composed of a multidisciplinary team of experts (Figure 1) who were mostly selected and invited by the MoHSW. Nearly two-thirds of the experts came from referral hospitals, specialised hospitals and the MoHSW, and these were mainly physicians and specialists. Half of them were female. Through document review we found that only three had been involved in the previous review of the STG/NEML. Two informants said they had received training on evidence-informed decision-making. One of these said:

*When I was pursuing my masters at […] we were at times being taught by people from foreign universities such as […] so they emphasised the use of evidence, particularly from meta-analyses in decision-making*. (Participant from Hospital)

#### The revision of the Standard Treatment Guidelines

At its first meeting, the secretariat discussed the approach to update the STG. A consensus was reached to split into groups according to medical specialities to simplify and speed up the revision process. Each group was tasked with revising a specific section of the guidelines pertaining to its speciality. One informant said:

*We started the review process by going through the old STG first; looking at what was missing, what to add and even what should be removed completely. We went through each disease condition one after another but not as a single panel. We were divided into specialities, people dealing with cancer looked at cancers, cardiology the same etc.* (Participant from Hospital)

The revision process varied between groups; some groups organised discussion meetings and disseminated their recommendations around their respective departments for comment, while in other groups the task was a take-home assignment for group members. The majority of informants said that the organisation, participation and time allocated to the task were not satisfactory.

*In my opinion the process is not perfect. First of all the time for review is very constrained, in such a way that you cannot have effective and detailed discussions on the management of patients and other important issues.* (Participant from Hospital)

*The process was so disorganised, I remember I presented the work of another person who was not there […]. I can say the whole organisation was not good.* (Participant from Hospital)

During the second meeting, group leaders presented the proposed recommendations to the secretariat so that other members could provide comments. Thereafter, these group works were compiled into a first draft of the STG. The PSS then extracted all the recommended medicines from this guideline to formulate the NEML. The draft of the STG/NEML was disseminated to different experts before it was submitted to the National Medicines and Therapeutic Committee (NMTC) for approval. One informant said:

*The draft of the STG/NEML was sent to the panel of reviewers for comment. Most of our reviewers come from major hospitals; we believe they have experience in research and publications. Then, after receiving their comments, we addressed them before we sent the document to the National Therapeutic Committee for approval.* (Participant from the MoHSW)

### Criteria used for Medicine Selection and the Underlying Evidence

The criteria employed and level of evidence varied between the groups updating different sections of the STG; some groups considered only one criterion while others used several. These criteria, together with their supporting evidence and other factors that influenced decision-making, are described below.

#### Efficacy and safety

A majority of informants said that they used an evidence-based approach in updating the STG/NEML. Efficacy and safety were the most cited criteria to have been used by different informants from the guideline review groups. Two informants said:

*We were using an evidence-based approach that a recommended drug must have shown that clinically it was more potent and produced more benefits and there is research evidence for that.* (Participant from Hospital)

*At the time of the review […] was a hot cake, people were trying to assess whether it was safe for patients. In general the concern was how it fares in the field as compared to […]; there were some discussions and we reached a consensus as you saw in the guidelines.* (Participant from Hospital)

Regarding the use of evidence, all informants acknowledged that evidence summaries for these criteria were not generated to inform decision-making, but claimed that such evidence was known to them through clinical experience. Two informants said:

*Clinical experience was crucial, because you want to produce a practical guideline. Therefore frankly speaking, a lot of evidence came from my daily practice and this was not scientific evidence but simply my experience.* (Participant from Hospital)

*Doctors were the ones who were recommending medicines for the Standard Treatment Guidelines to manage diseases through their clinical practice. Our belief was that as long as the medicine is being used in the hospital then automatically there was clinical evidence for that medicine to be selected.* (Participant from MoHSW)

The two informants who mentioned being trained in evidence-informed decision-making said that their recommendations were supported by research evidence from clinical trials and meta-analyses. However, during the interviews, as well as saying they did not develop evidence summaries, they were also not able to give sufficient explanation about how they searched and appraised the evidence. Some informants, particularly the pharmacists, recognised the lack of evidence in the decision-making process. One member of the secretariat expressed the following concern:

*We asked the physicians if the evidence they were giving to support their recommendations was actually based on scientific research! Unfortunately no one said it was scientific evidence. They all said the evidence was observation from their clinical practice and feedback from their patients.* (Participant from Hospital)

In some situations the interviewer challenged the informants with scientific evidence supporting the use of some medicines for a condition other than the one they had recommended. Surprisingly, some informants disagreed with such evidence, which was another indication of how difficult it was for scientific evidence to find its way onto the decision-making table compared with that from clinical experience. One such example is Tranexamic Acid (TXA) injection, a drug which reduces the risk of death in bleeding trauma patients [36]. In the STG it is recommended for prevention of mucosal bleeding. One informant said:

*I have never heard of any clinical trials done on tranexamic acid injection. I do not agree or believe in that research, it is not correct. Tranexamic acid is not a treatment, it is prevention and it is contraindicated in massive injuries. How can it help when someone has massive bleeding?* (Participant from Hospital)

#### Availability

All informants said that it is very important that the medicines they select are available on the Tanzanian market so that patients can access them even when they are not available at public healthcare facilities. They said that the existing policy requires all medicines in the STG/NEML to be written with generic names rather than brand names because generics are readily available, relatively cheap and affordable.

*We asked ourselves whether the medicines we were selecting were actually available in the market. The main question was: if a certain medicine in the list was prescribed will it be available in the Tanzanian market? We thought it would not make sense to have medicines in the STG/NEML which were not readily available in the country.* (Participant from Hospital)

Informants from the Tanzania Food and Drugs Authority (TFDA) said that the availability of medicines depends on whether they are registered in Tanzania or not. They said that in order for any medicine to be allowed to enter the Tanzanian market it has to go through a rigorous registration process in which its quality, efficacy and safety are thoroughly checked. They said that the TFDA registers all medicines that meet a minimum level of prescribed standards and it is from this pool that essential medicines are selected. One official explained:

*Drug registration actually involves many processes. In summary, the applicant, usually the manufacturer of the drug, must provide detailed information about the active pharmaceutical ingredient, the finished product and good manufacturing process which demonstrates the quality, safety and efficacy of the drug product.* (Participant from the TFDA)

None of the informants mentioned having used the list of registered medicines from the TFDA. Instead they said they knew the available medicines through their practice. Through the interviews we learned that healthcare workers in Tanzania frequently receive drug information from representatives from pharmaceutical companies. One informant said:

*We communicate a lot with our colleagues working with pharmaceutical companies, so they tell us if there are new drugs as first lines for certain diseases with better clinical outcomes.* (Participant from Hospital)

#### Affordability

A majority of informants said that affordability was an important criterion in medicine selection because economically Tanzania is very poor. Despite this concession, some informants said that there were disagreements between the doctors and pharmacists about the limit on the number of medicines to be added to the STG/NEML and whether expensive medicines should also be selected. Some doctors wanted the STG to have a variety of medicines from different therapeutic classes for each disease, some of which were considered expensive. Pharmacists, as the custodians of medicines, often opposed them because they were concerned about budget implications. There was no consensus about these disputes and the final ruling awaited the approval meeting of the National Medicines and Therapeutic Committee. One informant said:

*There were disagreements between me and the doctors, I told them essential medicine means to have a limited number of affordable medicines but they said “No! We are the ones who are in the field treating patients”. Therefore there is a need for people to be educated about the meaning of essential medicines.* (Participant from Hospital)

Informants said that they also took into account the total cost of treatment rather than the unit prices of individual medicines, especially for chronic diseases. One informant said:

*In some cases we looked at costs for a full course of treatment rather than unit prices. A month’s cost of a 50 Tanzanian shilling (Tshs) tablet taken three times a day is 4,500 Tshs compared to 3,000 Tshs for a once-a-day sustained-release tablet which costs 100 Tshs. You see, this is cheaper and increases compliance with treatment.* (Participant from Hospital)

Regarding the evidence for medicine prices, the majority said they knew the prices of most medicines through experience but some said they used price lists from the Medical Stores Department and drug representatives from private medicine suppliers. One informant said:

*So if two drugs were equally efficacious but one is cheaper then we selected the cheaper one so long as it has acceptable quality. We used the price list from the Medical Stores Department to compare the costs of the drugs.* (Participant from Hospital)

#### Cost-effectiveness

The understanding of cost-effectiveness analysis was poor among the majority of informants. Some confused it with cost comparisons and others were completely unaware of the concept. Those who said they were aware of it said that cost-effectiveness was not used as a criterion for medicine selection. They went on to say that economic evaluation studies are scarce in the country, and even if they were available they could not use them because they lack expertise.

Two informants said the following:

*Economic evaluation was not used at all. I think that means there should have been some studies about economic evaluation of medicines in Tanzania, which I am not sure if there is. Honestly, we have not taken on board such a criterion in the medicine selection process.* (Participant from the MoHSW)

*Well, that one I cannot say much about since it is not part of our expertise. I don’t remember in our group talking anything about economic evaluation of medicines, maybe in other groups but not the one I was with.* (Participant from Hospital)

Only one informant said he had received training about the use of economic evidence. Amongst the others, besides saying this was not an area in which they had expertise, one went further to comment that:

*Perhaps in the future, use of economic evaluation should be emphasised and the team involved in the review should be given lectures on pharmacoeconomics so that they can have knowledge about other criteria for inclusion of medicines in the Standard Treatment Guidelines besides clinical reasons.* (Participant from Hospital)

### Other Factors that Influenced Decision-making

#### International recommendations

Informants said that Tanzania has vertical programmes for HIV/AIDS, malaria, tuberculosis and leprosy. These programmes have their own guidelines, which are updated based on global recommendations. These recommendations were adopted in the STG. In addition, some informants said that they copied their recommendations from textbooks and guidelines from countries such as the USA, South Africa, Ghana and Lesotho. Regarding the use of international guidelines, one informant said:

*With malaria we follow the global recommendations, for example in 2010, the WHO malaria treatment guidelines were revised and artesunate injection was recommended for severe malaria. So it is the same with other ACTs, vaccines and some other medicines.* (Participant from MoHSW)

#### Promotion of medicines by the pharmaceutical industry

Some informants accused medicine promotion for influencing prescription practices. They were concerned that the medicines recommended for addition in the STG were there because of these types of influence. Two informants said:

*The second and most important point is that selection was the direct influence of medical representatives […]. They come here and talk to the doctors and they give them some free samples, what they get in return we do not know. With time doctors get used to these medicines and then they force their inclusion in the hospital formularies and later into the STG/NEML.* (Participant from Hospital)

*Lobbying by medical representatives is a problem. This hospital has a policy that medical representatives should make presentations in the meetings but sometimes they do not do that, they follow us into our offices to convince us to prescribe their products. They give gifts and some other things. Honestly, this is a common practice.* (Participant from Hospital)

To elaborate more on this practice, one informant said she used her experience and sometimes gathered evidence from journals. When she was asked to give the name of the journal, she said:

*Oooh! my Goodness I cannot remember the journal, but I can link you with those individuals at […] pharmaceutical companies and they can give you that information.* (Participant from Hospital)

In the informal interviews, drug representatives acknowledged that they persuade healthcare workers to procure and prescribe their products, and they do this by giving them free medicine samples and gifts such as stationery, refrigerators and televisions.

### The Approval of the STG/NEML

**Figure 3:**
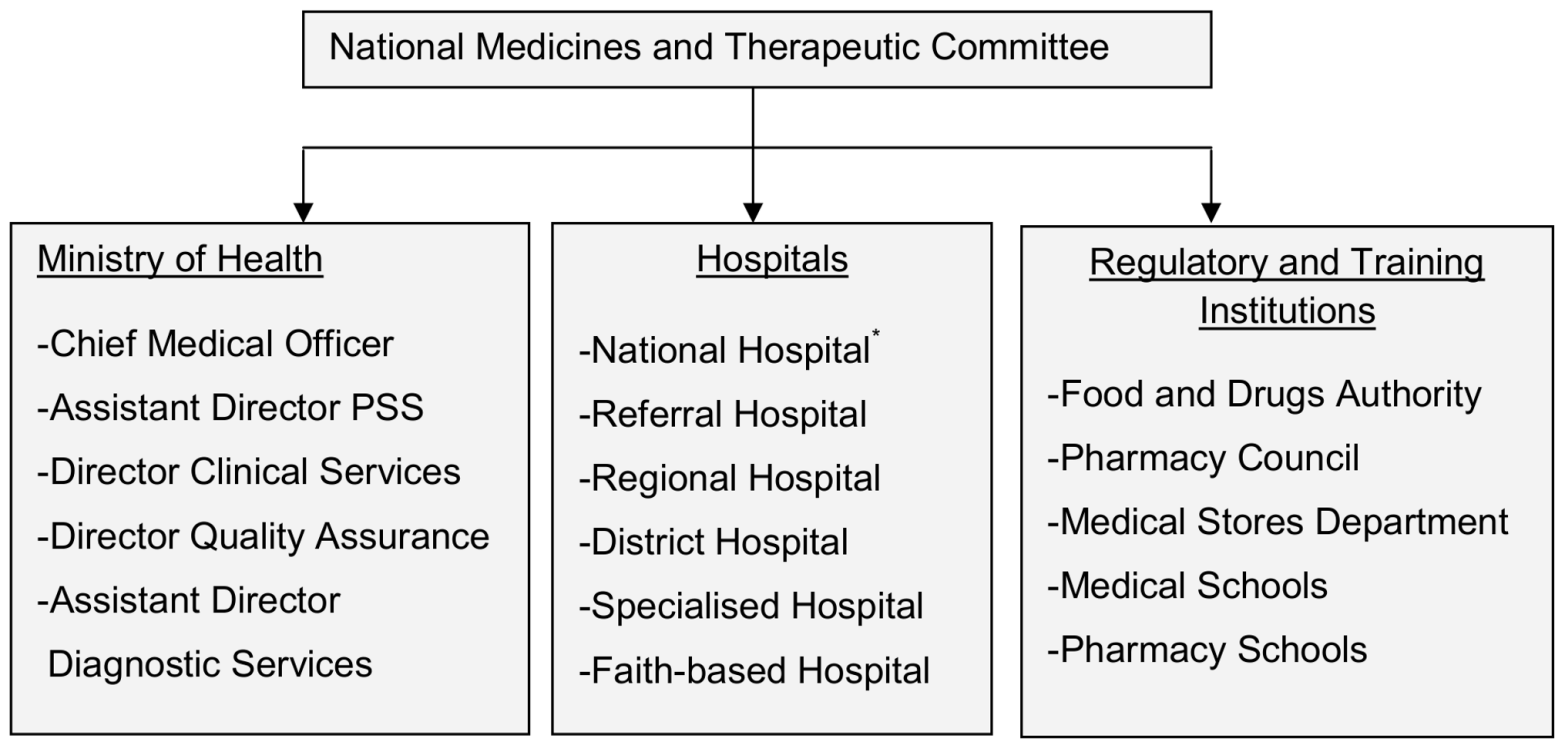
Composition of the National Medicines and Therapeutic Committee. *Three representatives.

The National Medicines and Therapeutic Committee (NMTC) is responsible for the approval of treatment guidelines and formularies in Tanzania and the PSS acts as the secretariat for this committee. The newly formed committee is multidisciplinary and consists of 18 members (Figure 3), with the Chief Medical Officer and the Assistant Director of PSS as the chairperson and secretary, respectively.

#### Process

The updated draft of the STG/NEML was submitted to the NMTC by the secretariat for approval in September 2012; about 130 new medicines were proposed to be added and five to be deleted. This draft was submitted without a summary of the changes and or the rationales behind them. Therefore the approval process proceeded first with the explanations given by the secretariat about the changes made in each chapter, followed by brief discussions. An excerpt from one of the document reads:

*The committee was taken through the reviewed STG and NEML, chapter by chapter. In each chapter presentations, the major changes which were made, in comparison to the STG/NEML edition of 2007, were explained to the panel. The NMTC members discussed and made recommendations.*

#### Criteria used in the approval process and use of evidence

There was no specific set of criteria used by the NMTC to approve each of the proposed changes. However, through document reviews, we found that the proposal to add clindamycin injection for the management of malaria in pregnancy was rejected because of safety and affordability concerns. The committee also ordered the secretariat to shorten the NEML, citing budget limitations as the only factor. This shows that to a certain extent some criteria were considered but again were not supported by evidence. One informant who participated in the approval process said:

*Is an evidence-based process used by the National Medicines and Therapeutic Committee at the moment? I don’t think so. Is the committee applying an evidence-based framework in decision-making processes? I don’t think that’s what is being done at the moment.* (Participant from the MoHSW)

The fourth edition of the STG/NEML was released in July 2013, and contained nearly all the medicines that were initially proposed for addition. In contrast to the previous editions, the new STG/NEML contains, as appendices, application forms for addition, deletion and change of dosage form, strength and indication of the listed medicines. The guiding criteria include efficacy, safety, cost-effectiveness, cost comparison and budgetary impact. These criteria must be supported by relevant evidence, such as the results of clinical trials conducted in Tanzania. An excerpt from the application form reads:

*Reasons why the proposed drug is preferred to drugs already in the NEML (Please attach not more than five supporting pieces of evidence with respect to efficacy, safety, cost, cost-effectiveness, others). State briefly the results of clinical trials conducted in Tanzania […]. If no official trials, state personal experience and/or submit documentary proof.*

## Discussion

The most important finding derived from this study is that essential medicines in Tanzania were largely selected through an experience-based process, in contrast to the evidence-based approach that was recommended by the WHO Expert Committee on Selection and Use of Essential Medicines in 2002 [13]. The use of an evidence-based approach has been documented as difficult to apply in developing countries [37], and this is consistent with our findings. The WHO Expert Committee usually publishes evidence supporting its decisions [38], hence decision-makers in developing countries can gauge the applicability of such evidence in their own context during medicine selection. We found that this opportunity was very rarely utilised in the case of Tanzania.

The review involved experts who were selected mostly from referral hospitals and the Ministry of Health and Social Welfare in a process that can be described as implicit and not sufficiently consultative. Participation by a wide array of stakeholders is important to ensure that the needs existing across all levels of the healthcare system are reflected in the STG/NEML. The effectiveness of guidelines is often compromised by the development process, and those guidelines that are imposed by higher levels have a high probability of being under-utilised or even rejected by healthcare workers [39].

We found that efficacy, safety, availability and affordability were the most commonly cited criteria employed in medicine selection. These criteria are to a large extent consistent with those recommended by the WHO [7]. Medicines to manage diseases such as malaria, HIV/AIDS, TB and leprosy, which are managed under vertical programmes, were adopted from international guidelines that usually employ the best available evidence. In addition, medical sales representatives were also considered to be influential in medicine selection as they are viewed as the main source of drug information for prescribers. Experience from East Africa shows that medicine promotion is widespread and poorly regulated and several studies have reported concerns about the influence of these sales representatives in medicine selection [40], [41], [42], [43].

The criterion of cost-effectiveness was not used despite being one of the most important criteria employed by medicine management committees in developed countries to inform formulary decisions [45], [46], [47], [48]. This finding is not surprising, considering the limited role of pharmacoeconomics in developing countries [49]. Several studies have cited the low availability of pharmacoeconomic studies in Tanzania [33], [42], [50] as the main barrier, but this study found that a lack of training could also be an important limitation. Studies have shown that, without training, decision-makers cannot understand, translate or apply economic evidence even when it is made available to them [47], [48].

Experience rather than scientific evidence played a major role in the decision-making processes. In the few cases where scientific evidence was claimed to have been used, there was neither a systematic search nor an appraisal of the evidence and evidence summaries were not generated to aid decision-making. Even for criteria such as availability and affordability, which are relatively easy to apply compared to efficacy and safety, evidence also mainly came from experience and not official sources such as the TFDA, Medical Stores Department or the International Drug Price Indicator Guide. This could be explained by the inexperience of the experts involved in the review process in using an evidence-based approach, the lack of guidelines on how to do the review and time constraints. Commitment to the use of research evidence and the availability of adequate infrastructures, tools and expertise are essential to facilitate evidence-informed decision-making [44].

### Strengths and Limitations

This study employed a qualitative approach, which is suitable for phenomena of which prior knowledge or understanding is limited [51], [52]. The method enabled participants to give detailed accounts of what they did, observed and experienced during the revision process, hence limiting the influence of any pre-conceived ideas held by the investigators. However, in-depth interviews have a tendency to introduce recall bias due to incorrect memorisation or failure to remember important aspects of the phenomenon under investigation [29], which was evident in our study. Recall bias was minimised because the study was conducted while the revision of the guidelines was still ongoing, albeit in the final stages.

The expertise of the principle investigator in essential medicine and the functioning of the medicine selection committees in Tanzania was instrumental in conducting this study. The potential influence of his prior experience on the interpretation and discussion of the findings was minimised through observing a well-accepted protocol for qualitative studies, and by involving co-authors in the analysis of the data, as well as in other phases of the study.

The committees were faced with many challenges in performing the reviews. Firstly, it appears that they were not given any training or guidelines about how to update the STG/NEML; secondly, the majority were performing a review for the first time; thirdly, they were not given sufficient time or other resources to carry out the review and, lastly, even the organisers were constrained by limited capacity and resources to carry out a smooth review process. Therefore we believe that all those who were involved performed the review of the STG/NEML to the best of their ability. This paper has thus identified areas that can be improved in future reviews.

### Conclusions

Recent decisions about the selection of essential medicines in Tanzania were made by committees of experts, who largely use experience and discretionary judgement, leaving evidence with only a limited role in decision-making processes. This practice increases the risk of adopting ineffective and costly interventions that may not be worth implementing. Because of this, the health authorities in Tanzania should take the necessary measures to ensure that limited health resources are allocated to proven interventions with the greatest potential to reduce the burden of disease and meet other public health goals. This can be achieved through the systematic application of relevant evidence-based criteria in priority-setting decisions between competing interventions. There may be several reasons for the current limited use of evidence in the decision-making process, but one hypothesis that remains to be explored is whether training experts in evidence-based decision-making would lead to a better and more explicit use of evidence.
